# Supplementary material for: Structural characterization of antibody-responses following Zolgensma treatment for AAV capsid engineering to expand patient cohorts
Source: Nat Commun. 2025 Apr 19;16:3731. doi: 10.1038/s41467-025-59088-4 (PMC12009303; doi:10.1038/s41467-025-59088-4)
Supplement: Supplementary file 1 — Supplementary Information [file 41467_2025_59088_MOESM1_ESM.pdf]

Structural characterization of antibody-responses following Zolgensma treatment for AAV capsid engineering to expand patient cohorts

Mario Mietzsch[1*], Jane Hsi[1], Austin R. Nelson[1], Neeta Khandekar[2], Ann-Maree Huang[2], Nicholas JC Smith[3], Jon Zachary[1], Lindsay Potts[1], Michelle A. Farrar[4,5], Paul Chipman[6], Mohammad Ghanem[7], Ian E. Alexander[2,8], Grant J. Logan[2], Juha T. Huiskonen[7], Robert McKenna[1*]

[1] Department of Biochemistry & Molecular Biology, Center for Structural Biology, McKnight Brain Institute. College of Medicine, University of Florida; Gainesville, Florida, USA.

[2] Gene Therapy Research Unit, Children's Medical Research Institute, Faculty of Medicine and Health, The University of Sydney and Sydney Children's Hospitals Network; Westmead, Australia.

[3] Discipline of Paediatrics, University of Adelaide, Women's and Children's Hospital, North Adelaide, South Australia, Australia; Department of Neurology and Clinical Neurophysiology, Women's and Children's Health Network, North Adelaide, South Australia, Australia

[4] School of Clinical Medicine, UNSW Medicine and Health, UNSW Medicine, Australia

[5] Department of Neurology, Sydney Children's Hospital, Randwick, Australia

[6] Interdisciplinary Center of Biotechnology Research, University of Florida; Gainesville, Florida, USA.

[7] Institute of Biotechnology, Helsinki Institute of Life Science HiLIFE, University of Helsinki; Helsinki, Finland.

[8] Discipline of Child and Adolescent Health, University of Sydney; Westmead, Australia.

* Correspondence: M.M.: mario.mietzsch@ufl.edu, R.M.: rmckenna@ufl.edu

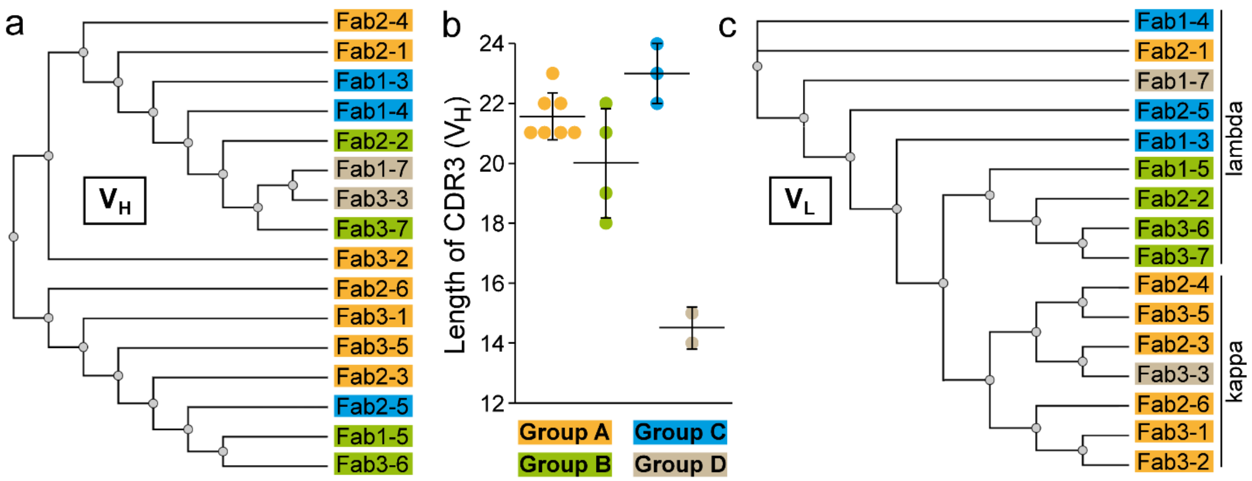

Supplementary Figure 1. Phylogenetic relationship of the 2-fold binding Fabs. **a)** A dendrogram of the Fab V_H chains was generated at https://ngphylogeny.fr/ using their primary amino acid sequence. The groups of the 2-fold Fabs are color-coded (orange = group A, green = B, blue = C, beige =D). **b)** The lengths of the $CDR3_H$ are plotted for each group (group A: n=7, group b: n=4, group C: n=3, group D: n=2). The data is presented as mean values +/- standard deviation (SD). **c)** Dendrogram as in (a) for the Fab V_L chains.

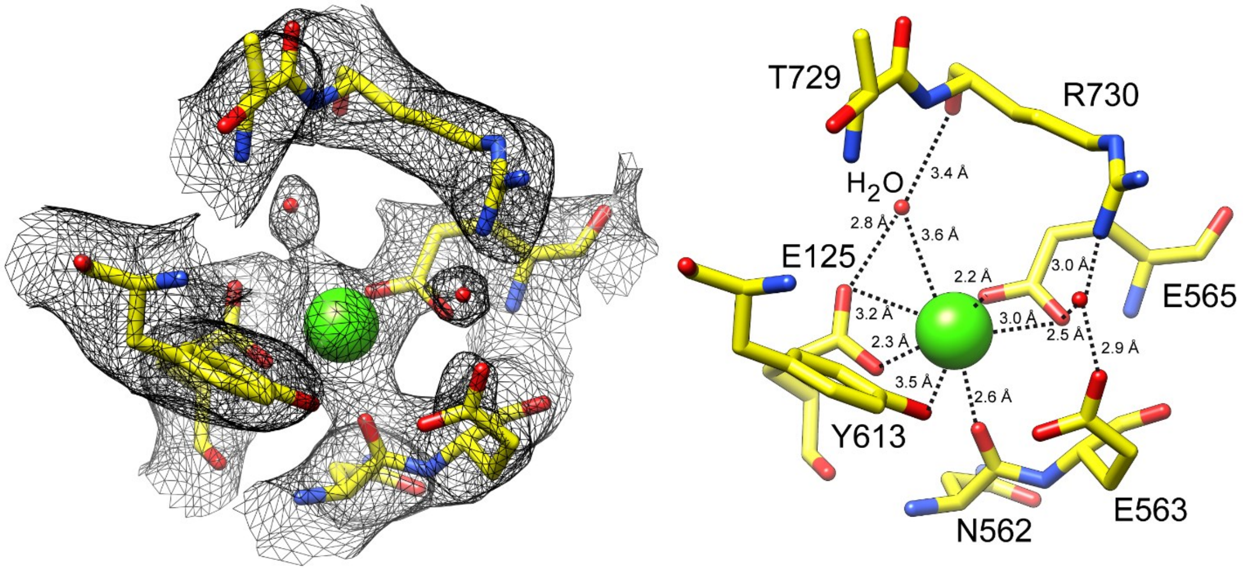

Supplementary Figure 2. Bivalent cation in the binding interface. A bivalent cation, e.g. calcium (green sphere), was identified in the binding interface of Fab2-3 (and Fab3-5) stabilized by multiple residues and two ordered water molecules. Left: The atomic model is shown inside the cryo-EM density map at a σ-threshold of 2. Right: The distances of the interactions in the model are provided. The amino acid residues are labeled and shown as stick representations and colored according to atom type: C = yellow, O = red, N = blue.

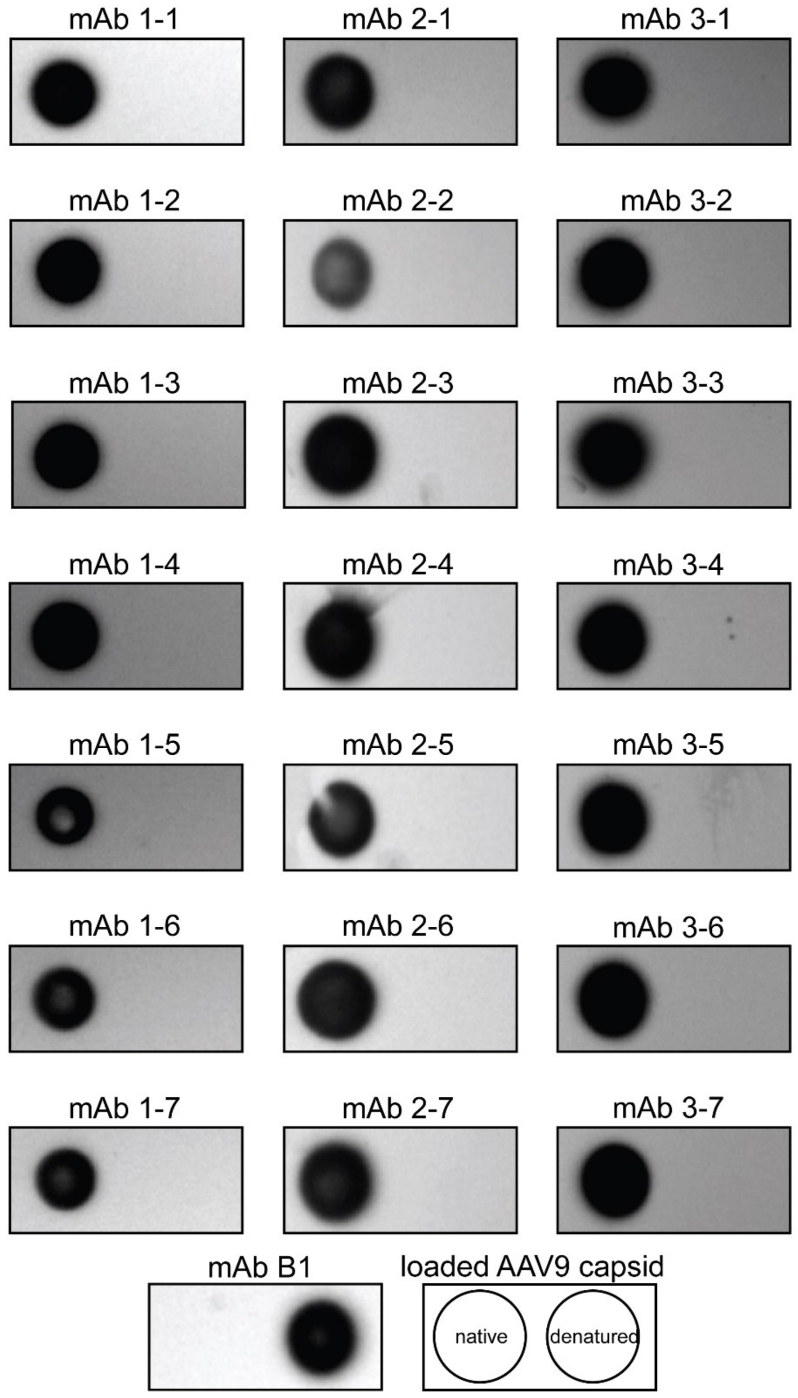

Supplementary Figure 3. The human mAbs only detect native capsids. Dot blots on $1x10^{10}$ AAV9 capsids loaded in their native condition and after incubation for 5 minutes at 95°C. The mAb B1 is used as a control which only detects denatured AAV capsids.

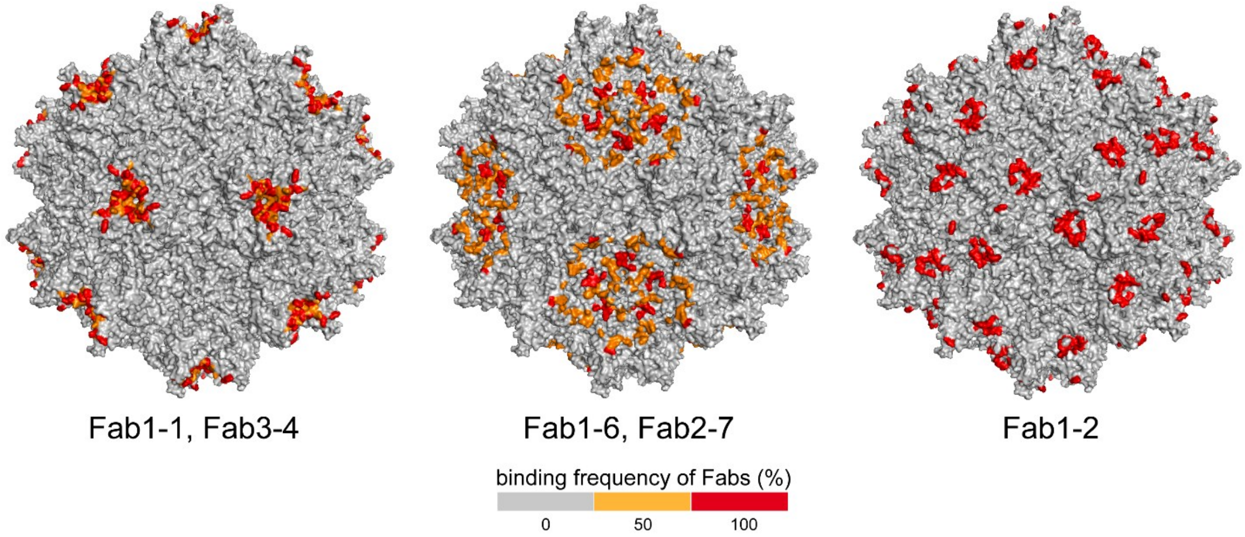

Supplementary Figure 4. Contact frequency of the Fabs to the AAV9 capsid. AAV9 capsid surface representation with the binding frequency for the 3-fold (left), 5-fold (center), and 2/5-fold wall (right) binding Fabs are displayed which are colored according to the bar below.

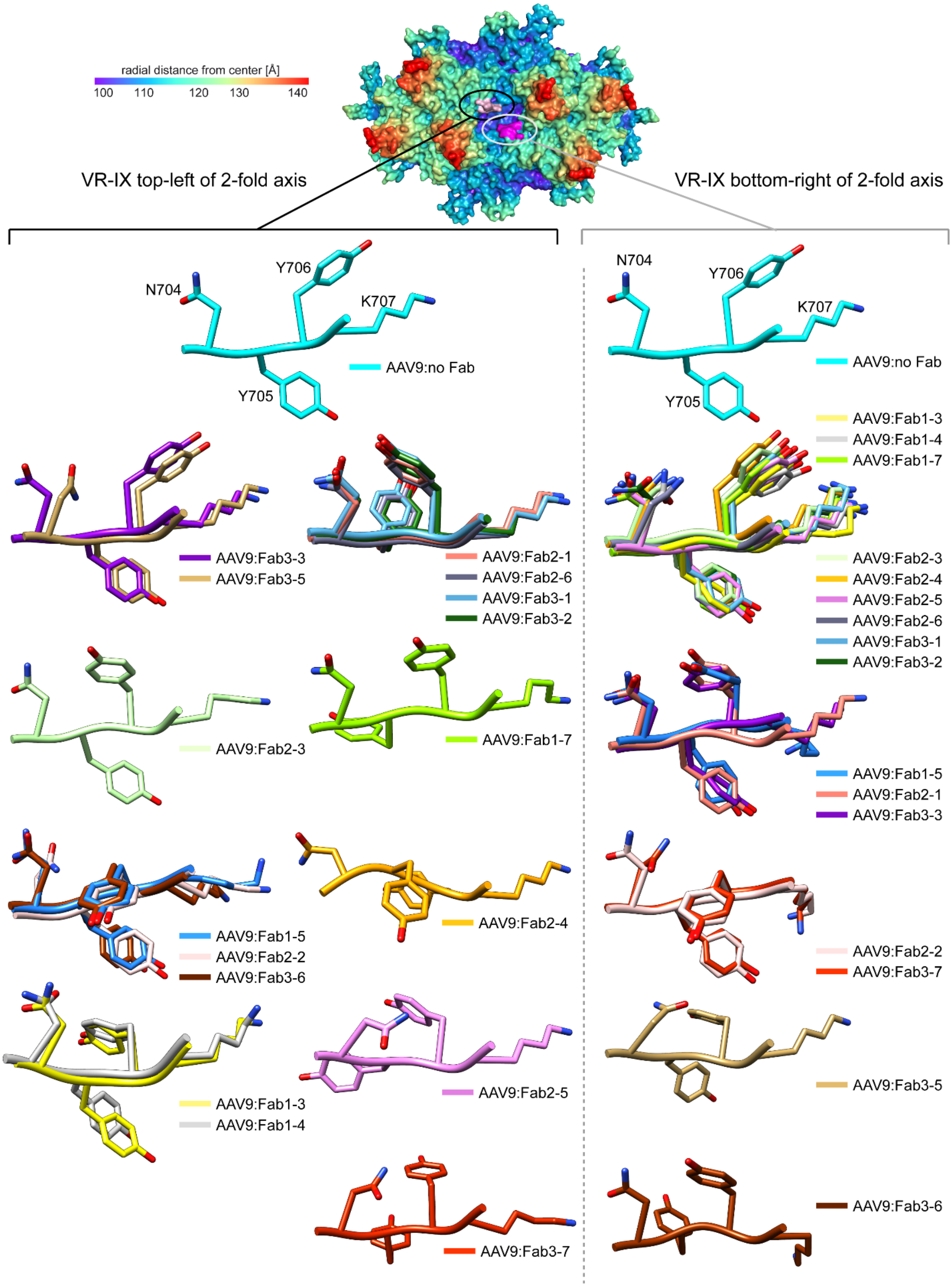
radial distance from center [Å]
100
110
120
130
140
VR-IX top-left of 2-fold axis
VR-IX bottom-right of 2-fold axis
N704
Y706
K707
Y705
AAV9:no Fab
AAV9:Fab3-3
AAV9:Fab3-5
AAV9:Fab2-1
AAV9:Fab2-6
AAV9:Fab3-1
AAV9:Fab3-2
AAV9:Fab2-3
AAV9:Fab1-7
AAV9:Fab1-5
AAV9:Fab2-2
AAV9:Fab3-6
AAV9:Fab2-4
AAV9:Fab1-3
AAV9:Fab1-4
AAV9:Fab2-5
AAV9:Fab3-7
AAV9:Fab2-3
AAV9:Fab2-4
AAV9:Fab2-5
AAV9:Fab2-6
AAV9:Fab3-1
AAV9:Fab3-2
AAV9:Fab1-5
AAV9:Fab2-1
AAV9:Fab3-3
AAV9:Fab2-2
AAV9:Fab3-7
AAV9:Fab3-5
AAV9:Fab3-6

Supplementary Figure 5. Fab induced conformational changes at the 2-fold region. A surface representation of the AAV9 capsid is shown for a double-trimer with the 2-fold symmetry axis in the center that is colored according to radial distance from the capsid's center (blue to red), as indicated by the scale bar. Atomic models of amino acid 704-707 adopt the same conformation in both symmetry-related VR-IX loops (colored pink and magenta in the surface representation) in absence of any Fab. In contrast, numerous variations of side-chain orientations for the residue range are observed following Fab binding. The amino acid residues are labeled and shown as stick representations with oxygen colored red and nitrogen blue.

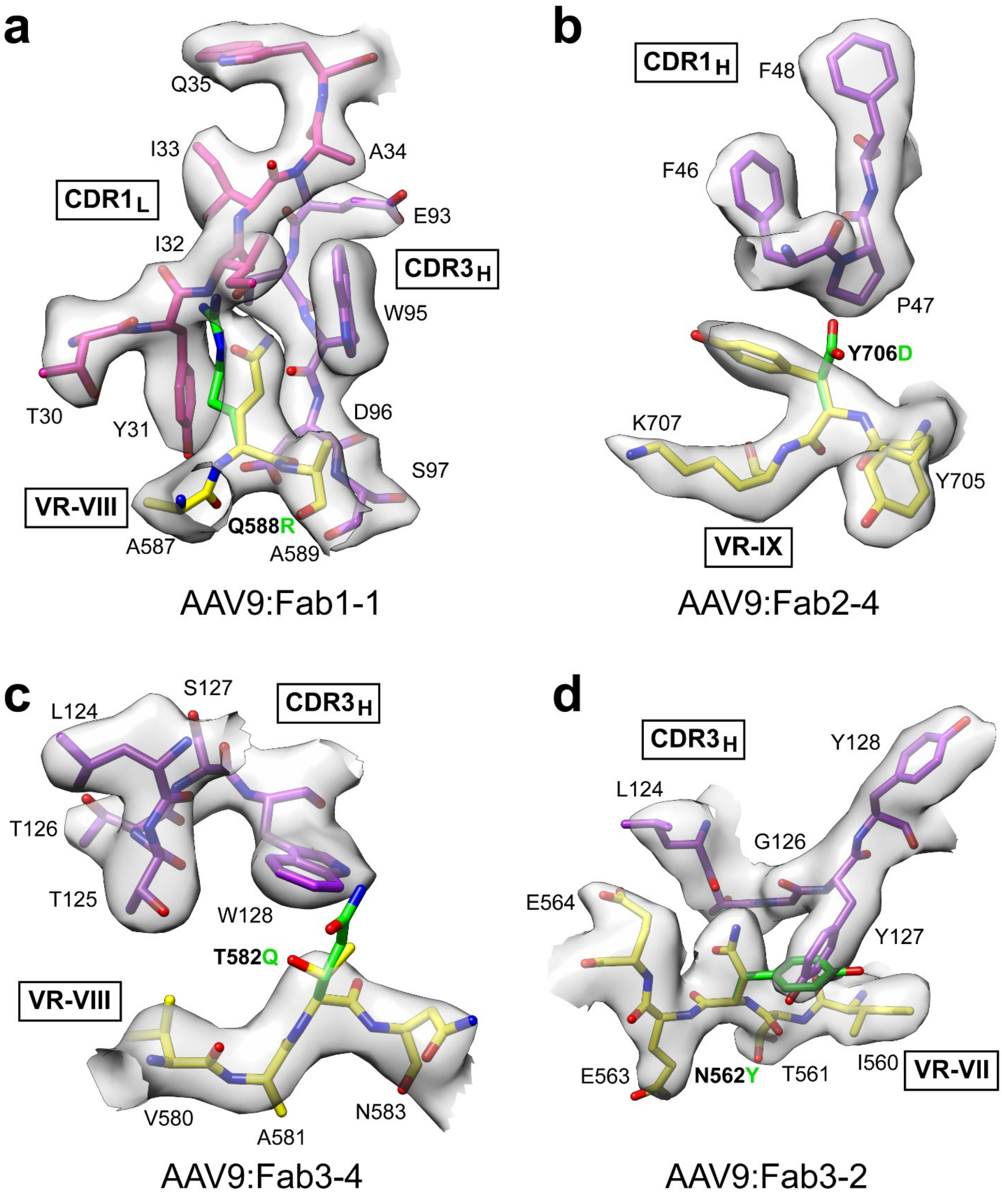

Supplementary Figure 6. AAV9-Fab contacts utilized for capsid engineering. **a)** Modeled AAV9-Fab1-1 interaction in VR-VIII (yellow), $CDR1_H$ (purple), and $CDR3_L$ (pink) inside the cryo-EM map. Substitution of Q588 to arginine (green) disrupts the interaction between capsid and

antibody. The amino acid residues are labeled and shown as stick representations with oxygen colored red and nitrogen blue. **b)** Modeled AAV9-Fab2-4 interaction in VR-IX, and $CDR1_H$ inside the cryo-EM map. The substitution of Y706 to aspartic acid (green) disrupts hydrophobic environment of the contact region. **c)** Modeled AAV9-Fab3-4 interaction in VR-VIII, and $CDR3_H$ inside the cryo-EM map. Substitution of T582 to glutamine (green) displaces the tryptophan on the Fab side and prevents Fab3-4 binding. **d)** Modeled AAV9-Fab3-2 interaction in VR-VII, and $CDR3_H$ inside the cryo-EM map. Substitution of N562 to tyrosine (green) clashes with $CDR3_H$ and prevents Fab3-2 binding.

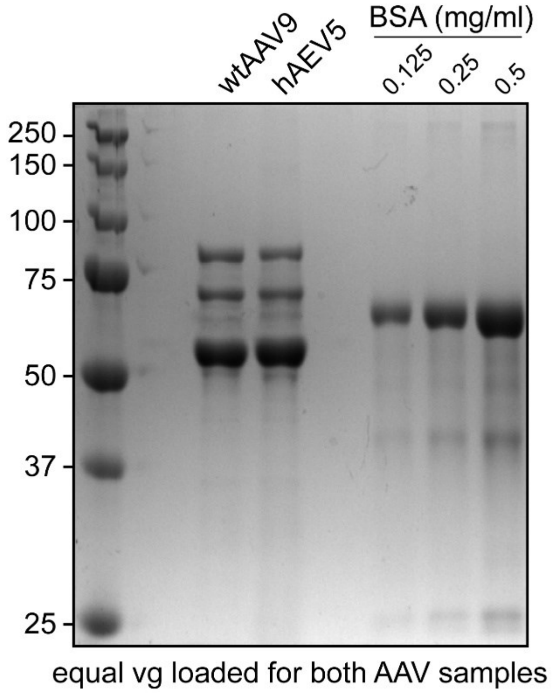

Supplementary Figure 7. Analysis of the empty-full ratio of AAV9 and hAEV5. An SDS-PAGE of purified AAV9 and hAEV5 vector samples is shown. Equal vector genomes of both samples were loaded onto the gel. Comparable VP bands intensity indicate similar empty-full ratios for either vector sample. The numbers to left represent the molecular weight (in kDa) of the bands from the protein ladder. Bovine serum albumin of known concentrations was loaded to estimate the protein concentration of AAV vector samples.

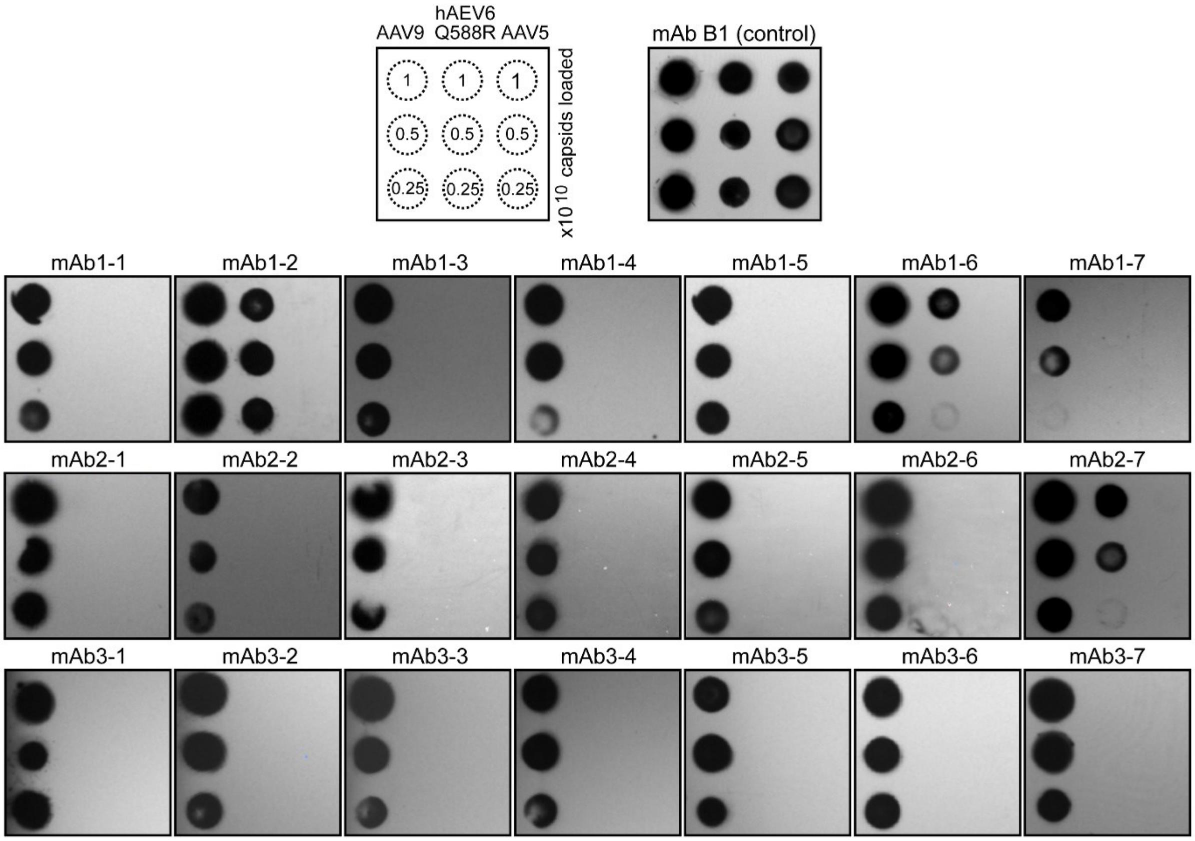

Supplementary Figure 8. Native immune-dot-blot of hAEV6-Q588R. Native dot blots are shown using mAb B1 as a loading control and the 21 human mAbs against the capsids of AAV9, hAEV6-Q588R, and AAV5. The hAEV6-Q588R capsid variant escapes 18 of the 21 human antibodies, except for mAb 1-2, 1-6, and 2-7.

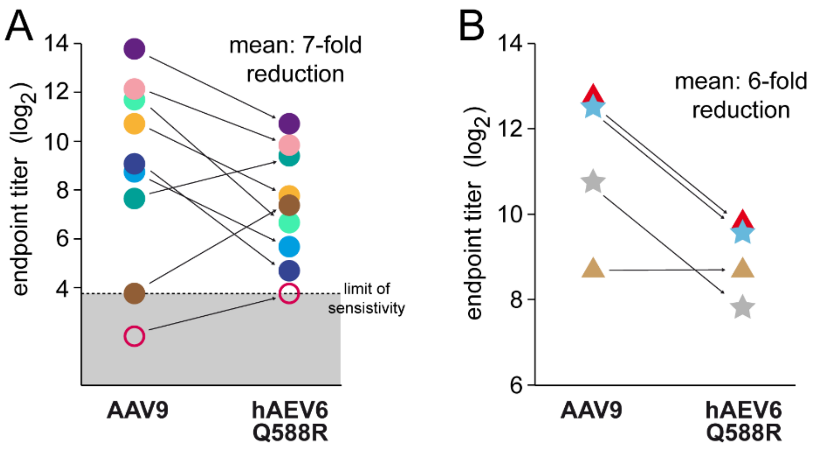

Supplementary Figure 9: Endpoint titer comparison of sera from non-Zolgensma recipients. **a)** Shown are the endpoint titers of AAV9-seropositive individuals (full circles) from a set of fifty healthy donors for AAV9 and the hAEV6-Q588R capsids. The individual patients are indicated as different colors. The limit of sensitivity (dilution < 1:12.5) is shown. An individual non-reactive to AAV9 capsid (open circle) shows weak reactivity to the hAEV6-Q588R capsid. **b)** Shown are the endpoint titers of children (triangles) precluded from an AAV9-mediated clinical trial for a childhood neurodegenerative disease and the mothers of infants (star-shaped) precluded from receiving Zolgensma for AAV9 and the hAEV6-Q588R capsids.

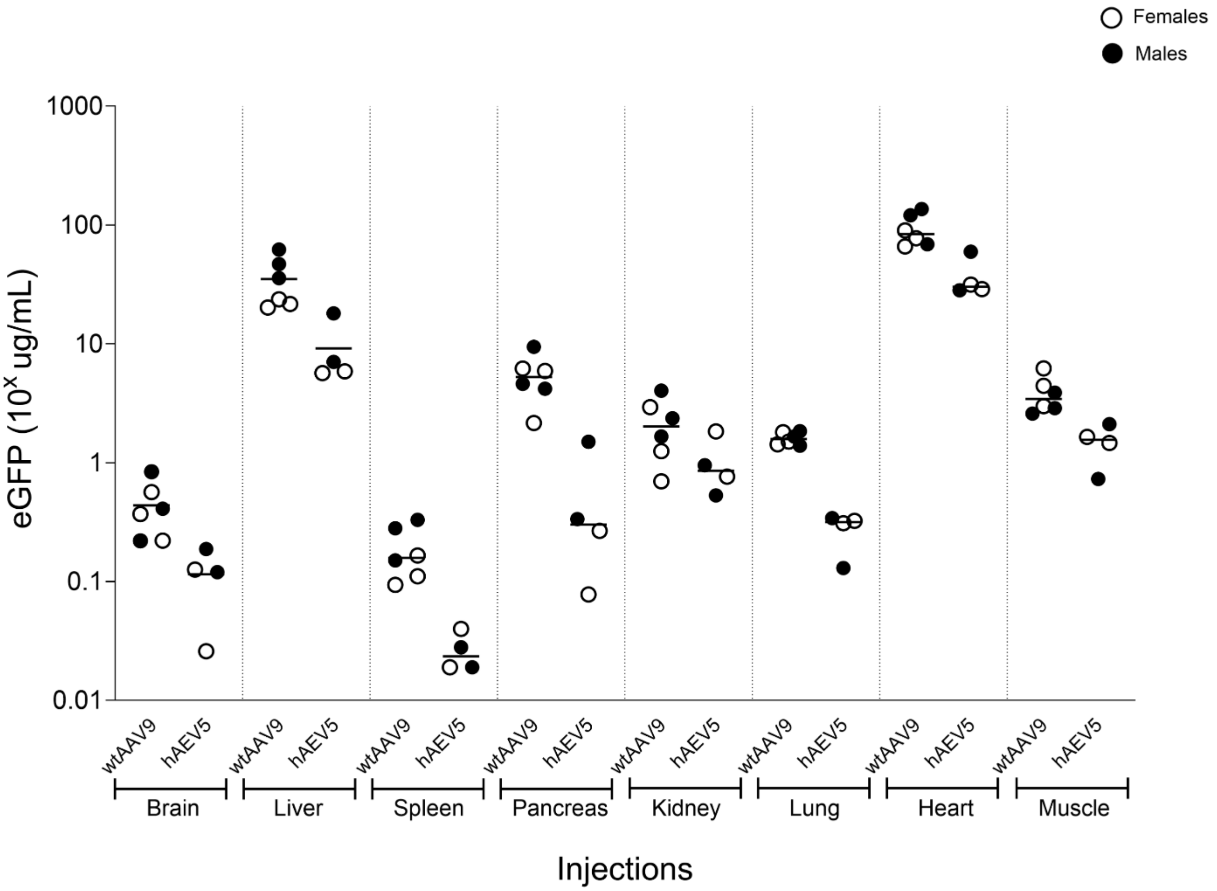

Supplementary Figure 10. Biodistribution of AAV9 and hAEV5. The GFP expression of AAV9 and hAEV5 vectors is shown for eight tissues following i.v. injection of 1.1 x 10^{14} vg/kg in C57BL/6 mice. Fully colored and open-colored circles represent male mice and female mice. The average of the data (n = 6 for AAV9 and n=4 for hAEV5) is indicated by the horizontal line.

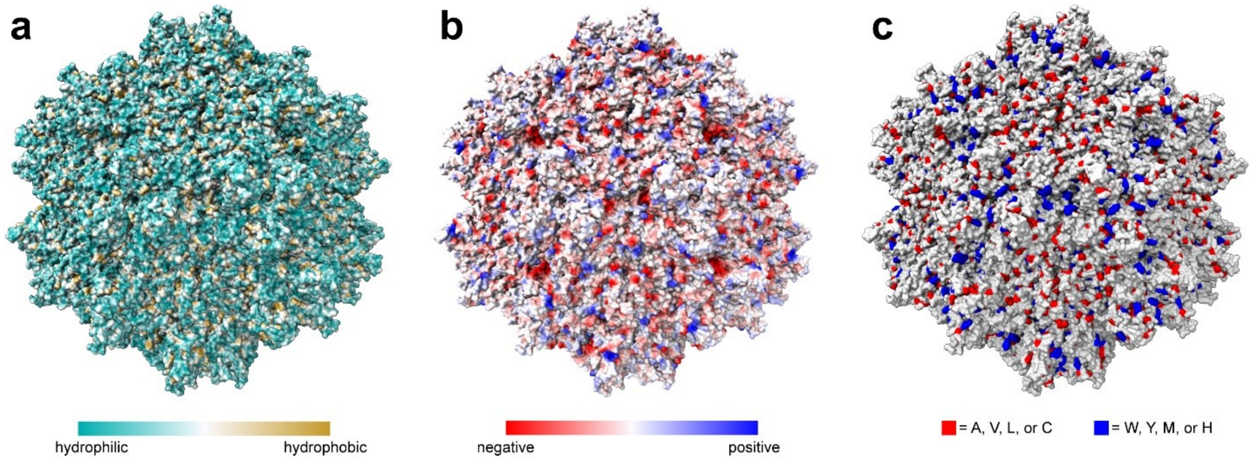

Supplementary Figure 11. AAV9 capsid surface. Surface representations of the AAV9 capsid are shown colored according to the scale bars below. **A)** The capsid is colored based on its hydrophobicity. **B)** The capsid is colored based on its electrostatic potential. **C)** Capsid surface residues alanine, valine, leucine, and cysteine are colored red, and tryptophan, tyrosine, methionine, and histidine are colored blue.

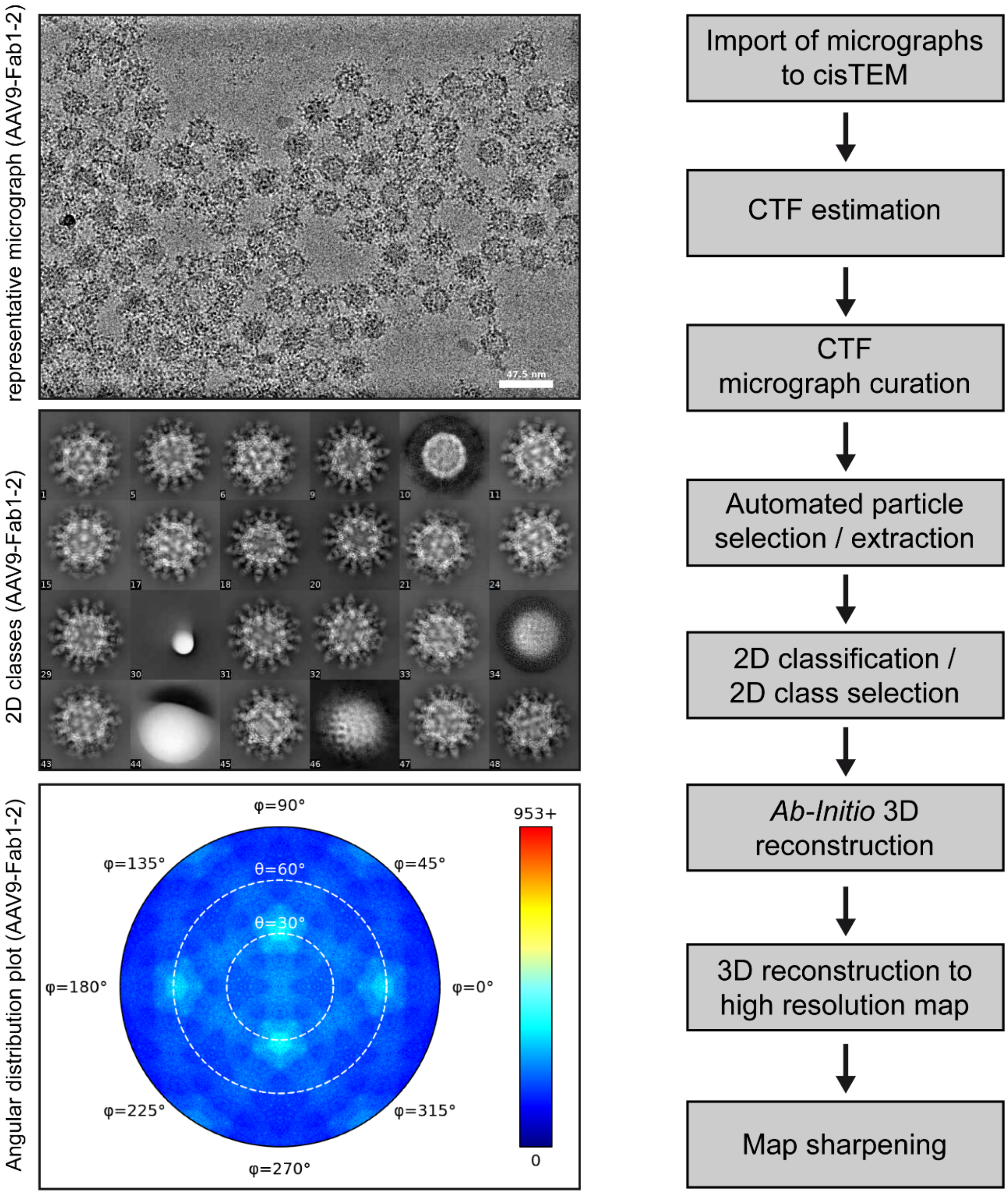

Supplementary Figure 12. Cryo-EM data processing workflow for Fabs binding icosahedrally to the AAV9 capsid. A representative micrograph as well as examples of the 2D-classes and an angular distribution plot of the particles used in the reconstruction are shown.

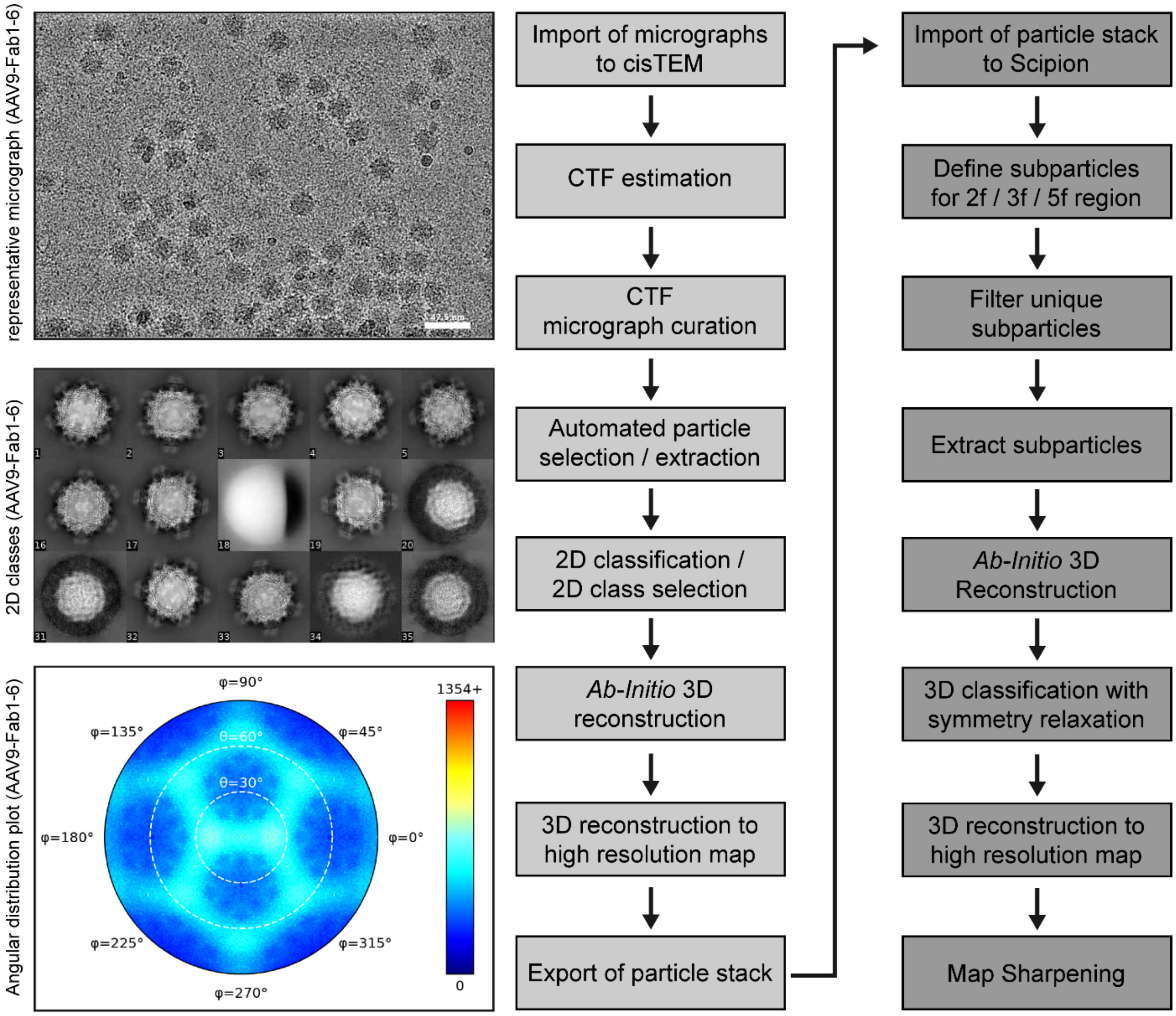

Supplementary Figure 13. Cryo-EM data processing workflow for Fabs binding in a non-icosahedral mode to the AAV9 capsid. A representative micrograph as well as examples of the 2D-classes and an angular distribution plot of the particles used in the reconstruction are shown.

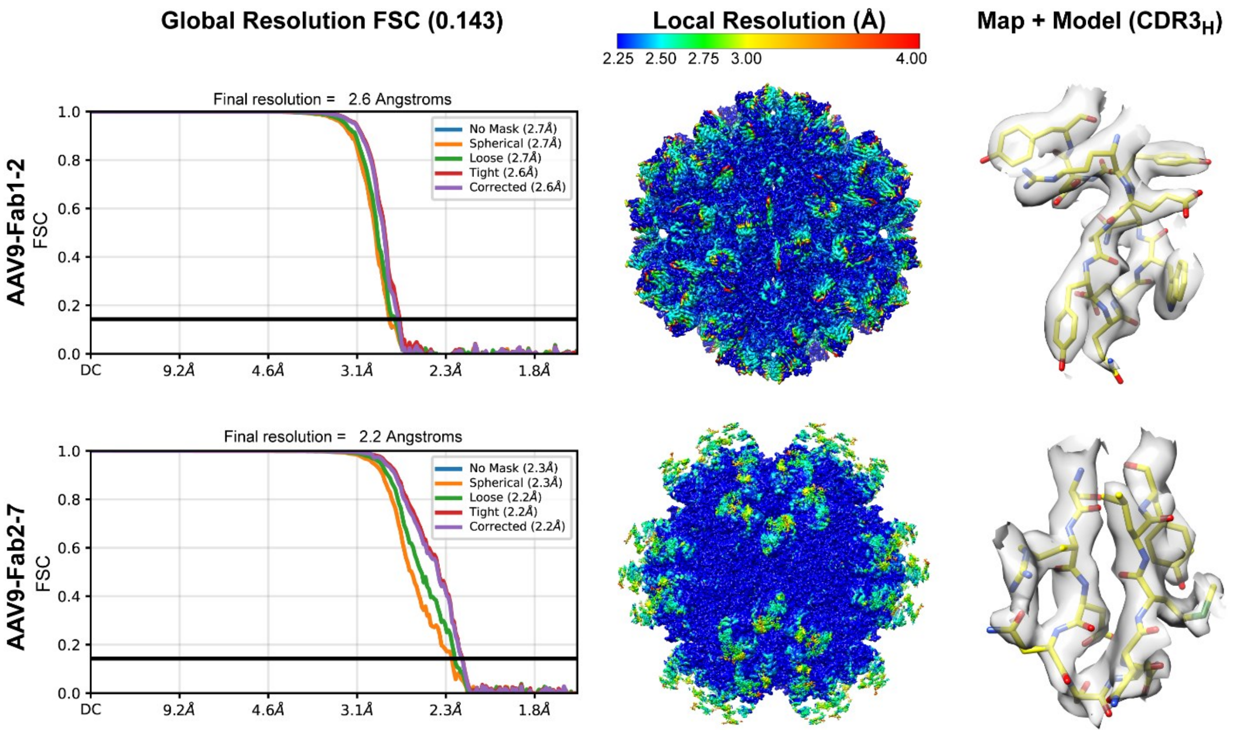

Supplementary Figure 14. Validation of the icosahedrally reconstructed cryo-EM maps. Shown are the global resolution estimates with FSC cut-off of 0.143, maps colored to local resolution based on the scale bar above, and model to map fit shown for the $CDR3_H$ region of the Fabs.

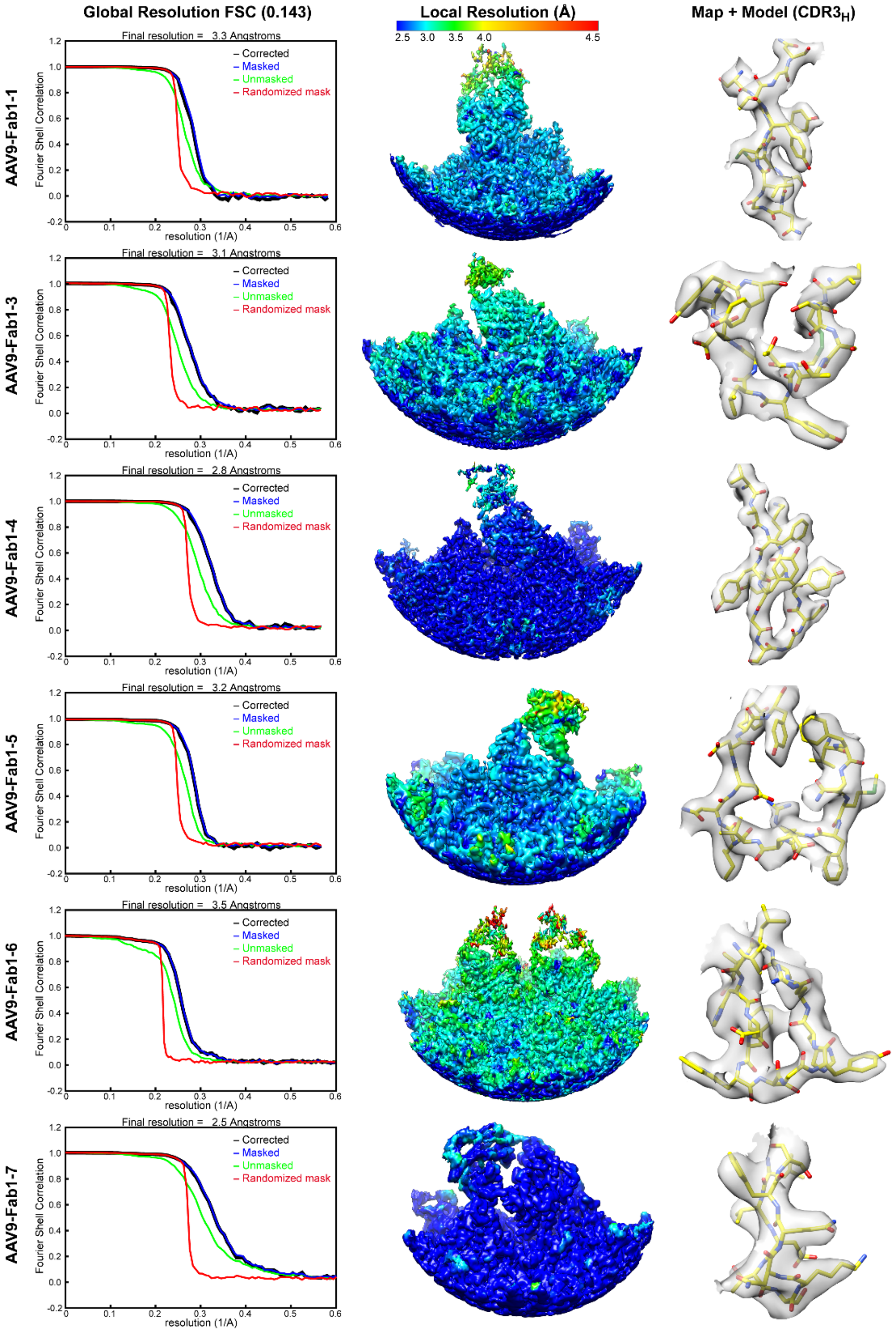

Supplementary Figure 15. Validation of the reconstructed cryo-EM maps for Fab1-1 to Fab1-7. Shown are the global resolution estimates with FSC cut-off of 0.143, maps colored to local resolution based on the scale bar above, and model to map fit shown for the CDR3$_H$ region of the Fabs.

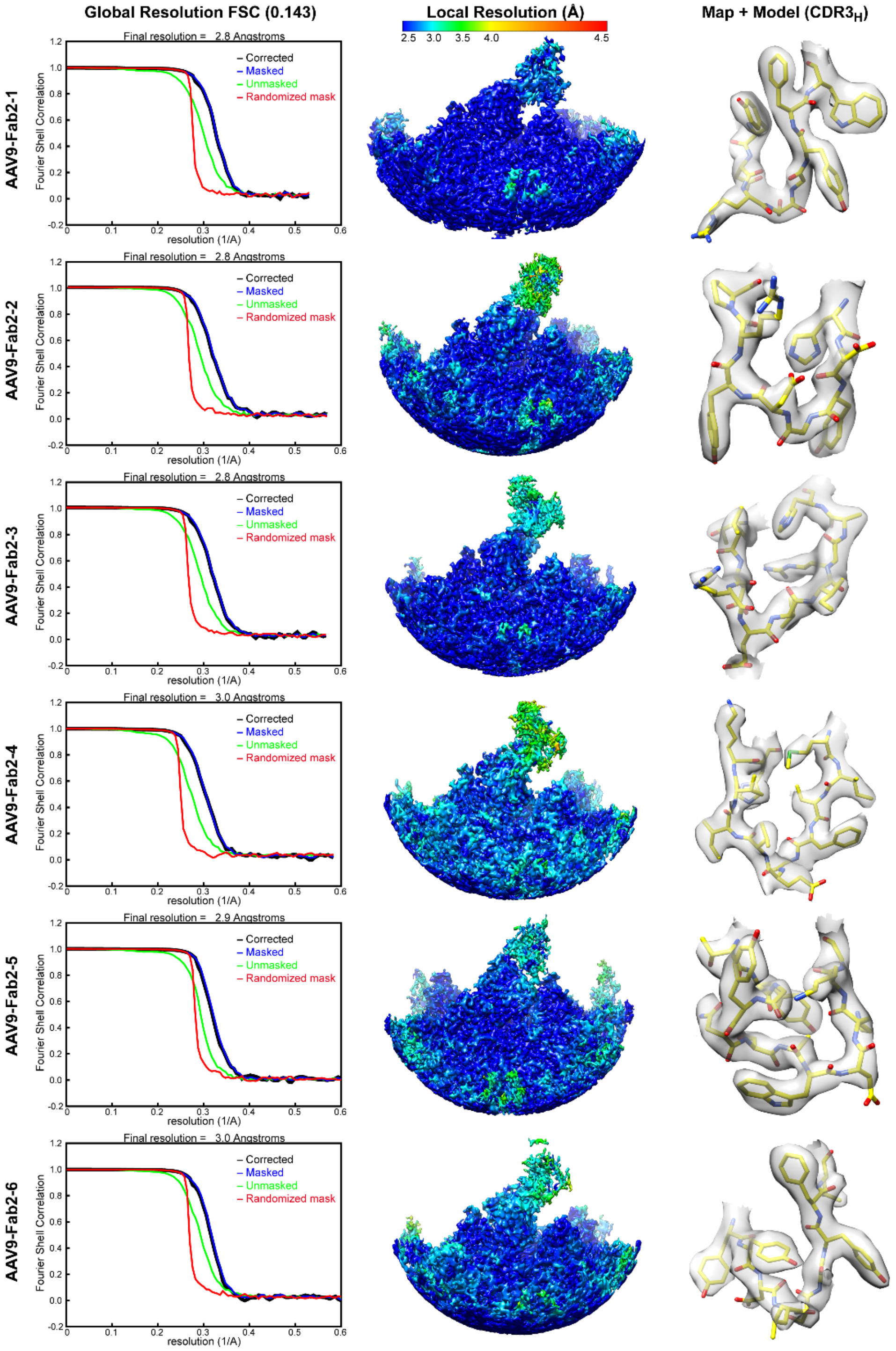

Supplementary Figure 16. Validation of the reconstructed cryo-EM maps for Fab2-1 to Fab2-6. Shown are the global resolution estimates with FSC cut-off of 0.143, maps colored to local resolution based on the scale bar above, and model to map fit shown for the CDR3$_H$ region of the Fabs.

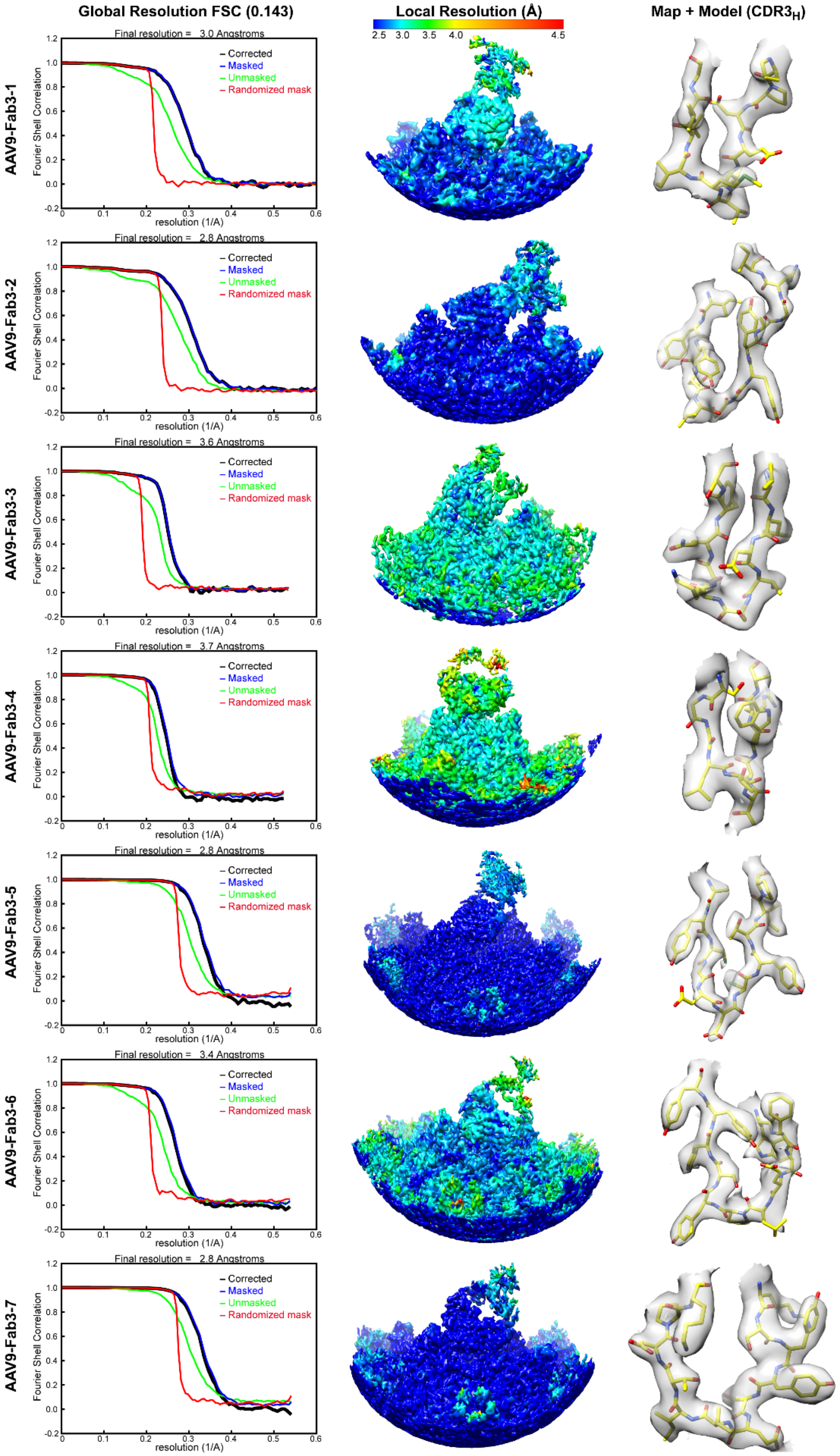

Global Resolution FSC (0.143)
Local Resolution (Å)
Map + Model (CDR3H)
2.5 3.0 3.5 4.0 4.5
AAV9-Fab3-1
Final resolution = 3.0 Angstroms
AAV9-Fab3-2
Final resolution = 2.8 Angstroms
AAV9-Fab3-3
Final resolution = 3.6 Angstroms
AAV9-Fab3-4
Final resolution = 3.7 Angstroms
AAV9-Fab3-5
Final resolution = 2.8 Angstroms
AAV9-Fab3-6
Final resolution = 3.4 Angstroms
AAV9-Fab3-7
Final resolution = 2.8 Angstroms
Corrected
Masked
Unmasked
Randomized mask
Fourier Shell Correlation
resolution (1/A)

Supplementary Figure 17. Validation of the reconstructed cryo-EM maps for Fab3-1 to Fab3-7. Shown are the global resolution estimates with FSC cut-off of 0.143, maps colored to local resolution based on the scale bar above, and model to map fit shown for the $CDR3_H$ region of the Fabs.

Supplementary Table 1: Summary of cryo-EM data collection, image processing, and refinement statistics.

AAV9-Fab complex	1-1	1-2	1-3	1-4	1-5	1-6	1-7	2-1	2-2	2-3	2-4	2-5	2-6	2-7	3-1	3-2	3-3	3-4	3-5	3-6	3-7
EMDB-ID	44271	44272	44273	44274	44275	44276	44277	44314	44315	44316	44317	44318	44319	44320	44321	44322	44323	44324	44325	44326	44327
PDB-ID	9B6N	9B6O	9B6P	9B6Q	9B6R	9B6S	9B6T	9B7K	9B7L	9B7M	9B7N	9B7O	9B7P	9B7Q	9B7R	9B7S	9B7T	9B7U	9B7V	9B7W	9B7X
**Data collection and processing**																					
Magnification	165K	165K	165K	165K	165K	165K	190K	190K	165K	165K	165K	165K	165K	165K	190K	190K	150K	150K	150K	150K	150K
Voltage (kV)	300																				
Defocus range (μm)	0.8 – 2.1																				
Electron exposure ($e^-/Å^2$)	50																				
Number of micrographs	4421	3790	7579	8998	5553	5831	5350	8791	7630	7170	7787	4767	2859	6232	5320	4731	4604	3180	3831	3226	3799
Pixel size (Å/pixel)	0.83	0.83	0.84	0.83	0.83	0.83	0.72	0.93	0.83	0.83	0.83	0.83	0.83	0.83	0.72	0.72	0.93	0.93	0.93	0.93	0.93
Capsid # icos. map	95K	173K	158K	151K	156K	246K	224K	366K	144K	148K	132K	78K	240K	263K	79K	52K	70K	112K	156K	44K	109K
Resolution icos. map (Å)	2.62	2.61	1.99	2.46	2.58	2.33	1.88	2.03	2.03	2.03	2.18	2.68	2.24	2.18	2.84	1.96	2.39	3.27	2.02	2.02	2.41
Subparticles # local. map	692K	N / A	411K	1.1M	284K	90K	428K	541K	1.1M	1.2M	361K	500K	2.1M	N / A	220K	80K	186K	623K	601K	481K	1.0M
Resolution local. map (Å)	3.31	N / A	3.13	2.77	3.19	3.47	2.54	2.75	2.82	2.82	3.02	2.86	2.98	N / A	3.02	2.84	3.56	3.73	2.76	3.36	2.76
**Refinement**																					
Initial model used (PDB)	3UX1																				
Model Res. $FSC_{0.5}$	3.2	2.7	3.4	2.7	3.5	3.7	3.0	2.9	2.8	2.8	3.0	3.0	3.1	2.6	3.3	3.1	3.7	3.7	2.8	3.5	3.0
Map CC	0.858	0.892	0.730	0.836	0.832	0.816	0.853	0.834	0.834	0.848	0.807	0.832	0.775	0.837	0.809	0.839	0.822	0.846	0.849	0.766	0.791
Map sharp. B factor ($Å^2$)	170	90	100	80	100	100	20	100	80	80	100	80	125	90	70	45	164	200	125	166	100
**Model composition**																					
Non-hydrogen atoms	13226	26548	17668	18765	17904	22854	12108	20567	19200	18744	18350	18072	14771	29725	9147	9273	18059	13874	20123	16963	19103
Protein residues	1658	3335	2247	2379	2275	2876	1550	2605	2434	2373	2323	2288	1865	3785	1167	1183	2282	1751	2546	2151	2424
**R.m.s. deviations**																					
Bond lengths (Å)	0.01	0.01	0.01	0.01	0.01	0.01	0.01	0.01	0.01	0.00	0.01	0.01	0.02	0.01	0.01	0.01	0.01	0.01	0.01	0.01	0.01
Bond angles (°)	0.80	0.85	0.75	0.79	0.73	1.01	0.80	0.86	0.86	0.69	0.74	0.70	0.85	0.96	0.80	0.83	0.81	0.68	0.81	0.89	0.80
**Validation**																					
MolProbity score	1.69	1.49	2.22	1.66	1.83	2.27	1.86	1.54	1.87	1.83	1.82	1.82	1.99	1.57	2.11	1.82	2.04	2.36	1.59	1.99	1.76
Clashscore	9.22	7.57	13.64	8.04	9.80	13.99	10.44	8.87	8.94	8.96	9.94	9.62	12.33	6.22	9.96	9.54	11.03	9.68	6.96	11.88	10.11
Poor rotamers (%)	0	0	0	0	0	0	0	0	0	0	0	0	0	0.2	0	0	0	0	0	0	0
**Ramachandran plot**																					
Favored (%)	96.8	96.5	94.6	96.5	95.4	95.0	95.7	96.6	96.4	95.4	95.9	96.6	95.8	96.3	95.4	96.0	94.9	95.9	96.5	94.8	96.7
Allowed (%)	3.2	3.5	5.3	3.5	4.6	4.7	4.3	3.4	3.6	4.6	4.1	3.4	4.2	3.7	4.5	4.0	5.1	4.0	3.5	5.1	3.3
Disallowed (%)	0	0	0.1	0	0	0.3	0	0	0	0	0	0	0	0	0.1	0	0	0.1	0	0.1	0

Supplementary Table 2: Contacts of the Fabs from patient 1 to the AAV9 capsid

			Fab1-1	Fab1-2	Fab1-3	Fab1-4	Fab1-5	Fab1-6	Fab1-7
Heavy Chain	CDR1	H-bonds	-	S49-Q387: 3.2<br>S49-N709: 3.2<br>N50-Q387: 3.1	*S47-N457: 3.2*<br>S47-Q458: 2.5	*T50-Q459: 3.5*<br>T50-T460: 2.8	S49-Q387: 3.3<br>*S50-K707: 2.7*	T49-N254: 3.3<br>*T49-N668: 3.0*	G45-N709: 3.1<br>Y51-D556: 2.5<br>Y52-T491: 2.8<br>Y52-T492: 3.0
		salt bridges	-	-	-	-	-	-	-
		other	-	-	G45-N457: 3.3<br>F46-N457: 3.4<br>S50-T460: 3.2<br>F51-V493: 2.4	*G45-Q456: 3.2*<br>*Y46-N457: 3.3*<br>Y52-D556: 3.2	M47-E712: 3.1<br>*S49-K707: 3.4*<br>S50-Y706: 3.1	-	Y52-R533: 3.3
	CDR2	H-bonds	K55-Q579: 2.7	N69-S265: 3.3<br>K71-S261: 2.3<br>Q72-S386: 2.7<br>*H78-S263: 3.3*	-	Q81-K707: 3.0	*R72-Y705: 3.4*<br>*R72-Y706: 3.5*<br>*R72-K707: 3.4*<br>S73-D384: 3.4<br>S75-E531: 2.8<br>*Y76-D532: 3.0*<br>*Y76-R533: 3.2*	S73-N254: 2.9<br>D76-T672: 2.9	S75-D532: 3.4<br>T92-N704: 3.5<br>S93-Y705: *3.1*/3.3
		salt bridges	-	-	-	D75-K557: 3.3	D78-R533: 3.7	-	-
		other	*K55-T593: 2.7*	Q72-N709: 3.2	Y72-D556: 2.5	-	Y76-E531: 2.8<br>Y76-R533: 3.3<br>Y76-F534: 2.9	*S73-F670: 2.9*<br>G74-T672: 2.6<br>A77-K332: 3.0<br>*Y79-G330: 2.4*<br>*V88-G330: 3.4*	S73-D532: 2.8<br>K94-Y706: 3.5<br>N95-Y706: 3.1
	CDR3	H-bonds	*N108-Q597: 3.1*<br>*G109-Q597: 3.0*<br>*C111-W595: 2.0 / 3.1*	Y120-D384: 2.7<br>Y120-E529: 3.3<br>Q121-N270: 3.0<br>*W123-G266: 3.0*<br>W123-N270: 3.0	S123-D532: 3.4<br>Y127-H527: 3.3<br>*Y127-F534: 2.7*	*P119-V493: 3.2*<br>N120-T492: 2.7<br>*V121-D556: 3.5*<br>Y124-H527: 3.0<br>Y125-K545: 3.3<br>Y125-N562: 3.1<br>G126-R725: 3.2<br>G128-R725: 2.9<br>N129-N704: 2.7 / 2.7<br>Y130-E712: 3.4<br>Y731-D532: 2.6	E126-Y731: 2.3<br>*I128-I560: 2.8*<br>*N129-A555: 3.0*<br>*N129-V558: 3.1*	*W122-S261: 3.2*<br>S123-Y274: 2.9<br>Y125-R550: 2.4	T119-D554: 3.0<br>*R124-Q459: 2.6*
		salt bridges	-	-	D130-K545: 2.7	-	-	-	-
		other	*L110-W595: 3.4*<br>*C111-G594: 3.2*<br>C111-Q597: 3.1<br>T113-W595: 3.1	Y120-K707: 3.3	Y127-F535: 3.2<br>G129-D556: 3.2	P119-T492: 3.3<br>*V121-T491: 2.8*<br>V121-V493: 3.3<br>*Y124-D532: 3.5*<br>Y125-M559: 3.4<br>Y125-P726: 3.5	F121-Y706: 2.9<br>*M123-Y705: 2.9*<br>F124-R391: 2.6<br>F124-Y731: 3.5<br>*V127-I560: 3.1*<br>I128-F535: 3.2<br>I128-N562: 3.3	*W122-I260: 3.5*	*V121-T491: 3.3*<br>V121-V493: 3.1<br>A122-T492: 3.4
Light Chain	CDR1	H-bonds	I32-Q588: 2.7 / 3.2	Y54-N270: 2.8	-	G50-N704: 3.4	Y52-D556: 3.1	*S46-G547: 3.0*<br>*G50-R550: 2.8*	N52-T494: 3.1
		salt bridges	-	-	-	-	-	-	-
		other	Y31-Q588: 3.3	Y54-G267: 3.5	-	S47-Y706: 3.3	-	S51-R550: 3.2	-
	CDR2	H-bonds	*S60-A591: 2.4*	-	N73-Y706: 2.6	Y70-Q495: 2.9<br>Y70-R533: 3.3<br>Q74-R533: 3.4	S74-Q387: 2.9<br>S74-N709: 2.4<br>R76-Q387: 2.6<br>*R76-K707: 2.9*<br>N82-N270: 3.5	R74-S469: 2.3<br>K87-N552: 3.4<br>*S88-N552: 3.3*	-
		salt bridges	-	-	-	-	R76-D384: 2.4	-	-
		other	G61-Q590: 3.1	L76-Q588: 3.0	K88-Y706: 3.2	K87-Y706: 3.2	F71-Y706: 3.0<br>N75-Y706: 3.0<br>N75-S708: 3.3<br>*G79-G530: 2.9*	Y70-T264: 3.2<br>K87-D551: 3.2<br>*D89-D551: 2.7*<br>*D89-N552: 3.5*	-
	CDR3	H-bonds	W95-Q588: 2.4<br>*D96-Q588: 3.2*<br>*S97-Q588: 3.3*	*S113-G267: 3.0*<br>*Y114-G267: 3.2*<br>Y114-S268: 2.4/3.0<br>S115-S268: 3.3<br>Y118-S265: 3.3<br>*Y118-G266: 3.5*	-	D114-N704: 3.3	S117-T492: 2.8	*R115-D657: 2.5*<br>*R115-S669: 3.5*	W112-T494: 2.8<br>N117-Q495: 2.7
		salt bridges	-	-	-	-	-	-	-
		other	-	*S113-G266: 3.2*<br>T115-S265: 3.3	S115-Y706: 3.1<br>S116-K707: 3.4	S115-Y706: 3.1	-	*R115-P658: 3.1*<br>R115-P659: 3.4	-
total contacts			18	26	17	33	39	27	20
interface area (Å^2)			1307	1734	1347	1778	2177	1779	1272
# VPs contacted			2	4	3	4	4	3	2

Supplementary Table 3: Contacts of the Fabs from patient 2 to the AAV9 capsid

			Fab2-1	Fab2-2	Fab2-3	Fab2-4	Fab2-5	Fab2-6	Fab2-7
Heavy Chain	CDR1	H-bonds	S49-Y705: 2.8	*S49-K707: 3.2*	-	N50-N704: 3.4	G45-N457: 3.4<br>S50-Q459: 2.9	S49-Y705: 2.5<br>Y51-N704: 3.2	
		salt bridges	-	-	-	-	-	-	-
		other	S49-Y706: 3.4	*S49-Y706: 3.4*	S49-Y706: 3.2	P47-N704: 3.5	Y51-V493: 3.4	G45-Y706: 3.3<br>S47-Y706: 3.3	
	CDR2	H-bonds	S71-D532: 2.5<br>S73-D532: 2.5<br>S76-D532: 2.7<br>N78-H_2O-T491 3.0/3.1	*Y71-G530: 3.4*<br>Y71-D532: 2.6<br>Y72-D384: 2.4<br>*S73-G530: 2.5*<br>N75-N495: 3.3<br>N75-R533: 3.3	*T76-G530: 3.4*<br>Y78-Q495: 2.9<br>Y78-R533: 3.1	S73-D532: 3.4<br>*G75-G530: 2.7*<br>G75-D532: 2.9<br>S76-D532: 3.3	S71-D556: 2.6<br>S73-D556: 2.9<br>Y76-K545: 3.0	S71-D532: 2.9<br>T76-D532: 2.8<br>N93-Y706: 3.5	S71-E718: 3.0<br>S73-T717: 3.1
		salt bridges	-	-	-	-	-	-	-
		other	*G75-G530: 3.4*	Y72-G530: 3.0<br>Y72-K707: 3.5<br>T92-Q387: 3.1	T76-R333: 3.5<br>N93-Y706: 3.2	Y93-Y706: 3.5<br>Y93-K707: 3.1	S73-D554: 3.1	*G74-G530: 3.4*	S76-E718: 3.4
	CDR3	H-bonds	Y121-N704: 3.5<br>R124-N562: 3.0<br>S125-N562: 3.3<br>*Y127-T561: 3.0*<br>*F128-D532: 2.9*<br>Y132-D556 3.2	Y121-K567: 3.5<br>G122-D532: 3.3<br>G122-N562: 2.4<br>Y124-H527: 3.5<br>Y124-F534: 3.3<br>Y124-H_2O-S540 2.8/2.5	R124-N562: 3.0<br>R124-H_2O-H527 2.9/3.0<br>*E125-N562: 2.7*<br>*E125-G728: 3.3*<br>E125-H_2O-R730 2.7/3.5<br>E125-Me^{2+}-E565-*N562* 2.3/2.6/*2.6*<br>*I127-I560: 2.9*<br>Y131-T492: 3.3	G124-N562: 2.6<br>E125-N562: 3.2<br>E125-Y731: 3.3<br>*I127-I560: 2.7*<br>Y131-T492: 2.9	*T121-T491: 2.9*<br>Y126-K545: 2.6<br>*N125-I560: 3.0*<br>G132-R533: 2.8	L124-N562: 3.1<br>G126-N562: 3.0<br>Y127-S526: 3.4<br>F128-T491: 3.3<br>*F128-D532: 3.3*	G127-D327: 3.2<br>S128-E324: 3.1<br>S128-T672: *3.1*/3.4<br>*T130-F670: 2.7/2.9*<br>T130-T672: 2.4<br>T131-S669: 3.5<br>N132-N254: 2.7/*3.1*<br>*N132-N668: 2.9*<br>Y134-K258: 3.2
		salt bridges	D123-R725: 2.6<br>R124-E529: 3.9<br>R124-D564: 3.3	-	D123-R725: 3.4<br>R124-D564: 3.4	E125-R725: 3.2	-	D123-R725: 2.6	
		other	Y121-N704: 3.2<br>R124-R391: 2.8<br>Y127-F534: 3.3<br>Y127-F535: 3.3<br>Y127-I560: 3.5<br>F128-T491: 3.0<br>W129-T491: 3.5<br>W129-A555: 3.3	Y121-D532: 2.7<br>*Y124-D532: 2.6*	R124-Y705: 2.8<br>*E125-P726: 3.2*<br>*V126-I560: 3.1*<br>V126-P726: 3.4<br>*I127-D532: 3.2*<br>*I127-R533: 3.5*<br>R128-T491: 3.4<br>A129-T491: 3.0	E125-P726: 2.9<br>*V126-I560: 3.2*<br>V126-P726: 3.4<br>*I127-D532: 3.3*<br>I127-N562: 3.3<br>K129-T491: 3.2	T120-T492: 3.2<br>T120-R533: 3.4<br>Y122-T491: 3.5<br>Y122-R533: 3.2<br>*N125-A555: 3.4*<br>*N125-V558: 3.2*<br>W128-P726: 3.2	Y122-D532: 3.4<br>L124-D564: 3.4<br>*Y126-D532: 3.2*<br>*Y126-F534: 3.2*<br>Y126-N562: 2.5<br>L129- T491: 3.3	I129-A656: 3.5<br>I129-S669: 2.7<br>I129-F670: 3.3<br>*T131-N668: 3.5*
Light Chain	CDR1	H-bonds	Y53-D556: 2.9	-	Q49-N457: 3.4<br>N52-D554: 3.4	-	Y53-D532: 2.7	-	N45-N552: 2.9
		salt bridges	-	-	-	-	-	-	-
		other	-	A51-D556: 3.2<br>Y52-D556: 3.1	-	Q47-N457: 3.2	-	-	-
	CDR2	H-bonds	-	S74-Q387: 2.9<br>N75-S708: 3.3<br>*R76-K707: 3.1*<br>R76-Q387: 2.7<br>S78-Y705: 2.5<br>S78-K707: 2.8<br>*G80-G530: 2.8*<br>N83-D384: 3.2<br>*N83-G385: 3.5*	S78-Q387: 3.2<br>S78-N709: 3.2	-	-	-	-
		salt bridges	-	R76-D384: 2.6	-	-	D71-R533: 2.9	-	-
		other	S77-Q387: 3.4	S74-N709: 3.4<br>*N75-K707: 3.4*<br>S79-D532: 3.0	-	-	K87-Y706: 3.3	-	-
	CDR3	H-bonds	-	*S118-V493: 3.4*	-	*Y114-Q459: 2.7*<br>Y114-T460: 3.4<br>S115-T494: 3.2	-	-	-
		salt bridges	-	-	-	-	-	-	-
		other	D114-V493: 3.4<br>D114-V494: 3.5<br>S117-T494: 3.5	S118-T492: 3.5	-	Y114-V493: 3.4<br>A116-T494: 2.8	S114-Y706: 3.4<br>S115-Y706: 3.4	-	-
total contacts			29	36	28	26	22	20	20
interface area (Å^2)			1630	1587	1567	1448	1347	1494	987
# VPs contacted			4	4	4	3	3	2	2

Supplementary Table 4: Contacts of the Fabs from patient 3 to the AAV9 capsid

			**Fab3-1**	**Fab3-2**	**Fab3-3**	**Fab3-4**	**Fab3-5**	**Fab3-6**	**Fab3-7**
**Heavy Chain**	**CDR1**	H-bonds	S49-Y705: 2.8<br>R50-N704: 2.8	S49-Y705: 2.9	S47-Y706: 2.9<br>S50-K545: 2.8<br>S50-P724: 3.3<br>S52-K545: 3.5<br>Y54-T492: 3.1/*2.8*	-	T47-N704: 2.7	S49-Q387: 2.8<br>*S50-K707: 3.1*	*S49-K707: 2.5*<br>*N50-K707: 3.3*
		salt bridges	-	-	H53-D556: 3.2	-	-	E53-R533: 3.3	-
		other	S49-Y706: 2.8	S49-Y706: 3.3	Y54-T491: 3.3	G45-Q588: 3.0<br>F46-Q588: 3.4	G45-Y706: 3.4<br>F46-Y706: 3.4	H47-Y706: 3.4<br>*S49-K707: 3.1*<br>*S50-Y706: 3.3*	N50-Y706: 3.0
	**CDR2**	H-bonds	S73-G530: 3.4	S71-D532: 3.2	S71-R533: 2.7<br>*S77-D532: 3.2*<br>S77-R533: 3.3<br>Y79-R533: 2.9	-	S71-D532: 3.0<br>G73-D532: 3.0<br>T75-D532: 2.5<br>Y77-Q495: 2.9<br>Y77-R533: 3.4<br>N92-N704: 3.5	Y69-R533: 2.7<br>S73-K707: 2.8<br>*T75-G530: 3.3*<br>*Y76-D532: 3.0*<br>*Y76-R533: 3.0*<br>N93-Q387: 3.3<br>P94-N709: 3.1	*Y72-G530: 3.4*<br>*S73-G530: 3.1*<br>S75-R533: 2.8
		salt bridges	-	-	-	-	-	-	-
		other	S71-R533: 3.1<br>Y78-T492: 3.3	N93-Y706: 3.3	Y73-D532: 3.1<br>L75-S532: 2.4<br>L75-N562: 2.6<br>S95-Y706: 2.9<br>N97-Y706: 3.4	F76-S499: 3.4<br>F76-Q590: 3.3<br>*F76-A591: 3.1*	S72-Y706: 3.3<br>N92-Y706: 3.4	*S73-G530: 3.2*<br>Y76-E531: 3.1	Y72-D384: 3.3
	**CDR3**	H-bonds	V124-N562: 3.5<br>T125-N562: 2.4<br>*I127-I560: 2.7*<br>V128-T491: 3.3	Y121-N704: 3.2<br>L124-N562: 3.1<br>*S125-P726: 3.5*<br>G126-N562: 3.4<br>Y127-S526: 3.3<br>*Y127-T561: 3.4*<br>G130-T492: 2.9<br>Y132-D556: 2.9<br>Y137-Y706: 2.8	-	*T125-W595: 3.5*<br>*T125-Q597: 3.5*<br>T126-N598: 3.5<br>*W128-W595: 2.4/2.6*<br>*R129-T593: 2.6*	C121-R725: 2.5<br>*D124-D562: 3.0*<br>*D124-G728: 2.6*<br>D124-Y731: 3.3<br>D124-Me^{2+}-E565-N562-D611<br>2.4/3.1/3.2<br>*Y126-F534: 2.9*<br>*Y126-I560: 2.8*	E123-N704: 2.6<br>P125-N562: 3.1<br>*Y126-F534: 2.5*<br>Y128-D532: 2.3<br>Y129-N704: 2.9	S120-Y705: 2.7<br>S120-Y706: 3.1<br>Y122-T702: 2.9<br>Y122-N704: 3.4<br>Y122-Y705: 3.2<br>N123-N562: 3.0<br>N123-E564: 2.9<br>E124-N562: 3.0<br>*E124-G728: 3.0*<br>E124-Y731: 2.5<br>*I126-I560: 3.0*
		salt bridges	D122-R533: 3.2<br>E123-R725: 2.8	D123-R725: 3.2<br>R131-D556: 2.9	-	-	-	-	-
		other	*T125-P726: 3.1*<br>*M126-I560: 3.3*<br>M126-P724: 3.0<br>M126-R725: 2.7<br>M126-P726: 3.2<br>I127-D532: 3.2<br>I127-N562: 2.9<br>V128-R533: 2.9<br>M131-D556: 3.1	Y121-R725: 3.4<br>Y122-Y705: 3.4<br>L124-E564: 3.4<br>*Y127-D532: 3.1*<br>*Y127-F534: 3.1*<br>Y127-F535: 3.4<br>Y127-N562: 2.7<br>Y128-T491: 2.9<br>*G130-T491: 3.1*	-	T125-N598: 3.0<br>T126-W595: 3.3<br>T126-V596: 2.8<br>T126-N598: 2.8/2.8<br>S127-V580: 3.4<br>S127-W595: 3.3<br>S127-Q597: 2.9<br>W128-T582: 3.0<br>W128-Q597: 3.4<br>W132-Q592: 2.9	D124-T561: 3.1<br>C125-I560: 2.8<br>C125-P726: 3.4<br>Y126-S526: 3.0<br>Y126-H527: 3.1<br>*Y126-D532: 2.8*<br>Y126-F535: 3.4<br>Y126-D562: 2.7	F121-Y706: 3.4<br>E123-Y705: 3.1<br>P125-E564: 2.4<br>*Y126-D532: 3.0*<br>Y126-F535: 3.2<br>*Y126-I560: 3.5*<br>Y126-N562: 2.9	R119-N704: 3.0<br>R119-Y706: 3.2<br>Y122-S703: 3.2<br>E124-P726: 2.4<br>V125-I560: 3.2<br>V125-P726: 3.5<br>*I126-D532: 2.9*<br>I126-D562: 2.9
**Light Chain**	**CDR1**	H-bonds	-	-	N52-N498: 2.8<br>S53-Q458: 3.1<br>W54-Q459: 2.8	-	Q49-N459: 3.3	-	Y54-D556: 2.9
		salt bridges	-	-	-	-	R53-D554: 3.8	-	-
		other	-	-	-	-	-	-	-
	**CDR2**	H-bonds	-	T78-Q387: 3.2	S72-Q458: 2.3	N74-Q590: 2.9	-	S74-Q387: 2.9<br>S74-N709: 3.5<br>*R76-K707: 3.0/3.2*<br>S78-D532: 3.5<br>S78-K707: 3.4<br>*N82-G385: 3.2*	S74-Q387: 2.9<br>S74-N709: 3.4<br>*N75-K707: 3.2*<br>R76-Q387: 2.6<br>*R76-K707: 3.3*<br>N82-D384: 2.8
		salt bridges	-	-	-	-	-	-	R76-D384: 2.6
		other	-	-	-	-	-	N75-Y706: 2.5<br>R76-D384: 3.3<br>*G79-G530: 3.0*	N75-S708: 2.3<br>*G79-G530: 3.5*<br>*N82-G385: 3.0*
	**CDR3**	H-bonds	Y115-N457: 3.5<br>Y115-Q459: 2.3<br>W116-T492: 3.1<br>W116-T494: 3.0	-	A113-T494: 3.3<br>N114-T494: 2.7	*S116-Q588: 2.9*<br>S116-Q590: 2.6	*Y114-N457: 3.3*<br>*Y114-Q459: 2.9/3.0*<br>I116-T494: 3.3/3.4<br>I116-Q495: 3.4	S117-T492: 3.5<br>*R115-N457: 3.4*<br>*R115-Q459: 2.9*	-
		salt bridges	-	-	-	-	-	-	-
		other	P117-T494: 2.9	-	F116-Q495: 3.1	-	Y114-T494: 3.0<br>S115-T494: 3.3	-	-
total contacts			26	25	24	25	36	40	37
interface area (Å^2)			1497	1367	1320	1280	1452	2068	2001
# VPs contacted			3	3	2	3	3	5	4

Supplementary Table 5: Capsid variants tested against Fab1-2, Fab1-6, and Fab2-7

Capsid variant	Fab1-2	Fab1-6	Fab2-7
N254H	not tested	does not escape	does not escape
K258R	does not escape	does not escape	does not escape
S261A	---- Capsid variant not infectious ----		
N262T	does not escape	not tested	does not escape
T264A	---- Capsid variant not infectious ----		
G266S	---- Capsid variant not infectious ----		
G267S	---- Capsid variant not infectious ----		
Ins-A267	---- Capsid variant not infectious ----		
N270Q	---- Capsid variant not infectious ----		
D384Q	---- Capsid variant not infectious ----		
S386R	---- Capsid variant not infectious ----		
D441N	does not escape	does not escape	does not escape
A502N	---- Capsid variant not infectious ----		
G530E	does not escape	not tested	does not escape
G530N/D532N	does not escape	not tested	does not escape
R533S	does not escape	not tested	does not escape
G549Q	does not escape	does not escape	does not escape
R550S	---- Capsid variant not infectious ----		
N552A/V553A	does not escape	does not escape	does not escape
I560F	does not escape	not tested	does not escape
A656Q	---- Capsid variant not infectious ----		
A656G/P658L	not tested	does not escape	not tested
S669Q	not tested	does not escape	not tested
N704Q	---- Capsid variant not infectious ----		
Y706D/N709I	does not escape	does not escape	does not escape

Non-infectious capsid variants showed transduction efficiencies <10% vs. wtAAV9 capsids

Supplementary Table 6: Primer sequences

Primer name	Primer sequence (5'-3')
N254H-Fwd	CTGCCCACCTACAACCATCACCTCTACAAGC
N254H-Rev	GCTTGTAGAGGTGATGGTTGTAGGTGGGCAG
S261A-Fwd	CCTCTACAAGCAAATCGCCAACAGCACATCTGG
S261A-Rev	CCAGATGTGCTGTTGGCGATTTGCTTGTAGAGG
T264A-Fwd	GCAAATCTCCAACAGCGCATCTGGAGGATCTTC
T264A-Rev	GAAGATCCTCCAGATGCGCTGTTGGAGATTTGC
G266S-Fwd	CTCCAACAGCACATCTAGCGGATCTTCAAATGACAAC
G266S-Rev	GTTGTCATTTGAAGATCCGCTAGATGTGCTGTTGGAG
G267S-Fwd	CCAACAGCACATCTGGAAGCTCTTCAAATGACAACG
G267S-Rev	CGTTGTCATTTGAAGAGCTTCCAGATGTGCTGTTGG
Ins-A267-Fwd	CCAACAGCACATCTGGAGCAGGATCTTCAAATGACAACG
Ins-A267-Rev	CGTTGTCATTTGAAGATCCTGCTCCAGATGTGCTGTTGG
N270Q-Fwd	CATCTGGAGGATCTTCACAGGACAACGCCTACTTCG
N270Q-Rev	CGAAGTAGGCGTTGTCCTGTGAAGATCCTCCAGATG
D384Q-Fwd	GTATCTGACGCTTAATCAGGGAAGCCAGGCCGTG
D384Q-Rev	CACGGCCTGGCTTCCCTGATTAAGCGTCAGATAC
S386R-Fwd	GCTTAATGATGGAAGGCAGGCCGTGGGTC
S386R-Rev	GACCCACGGCCTGCCTTCCATCATTAAGC
D441N-Fwd	CTAATGAATCCACTCATCAACCAATACTTGTACTATCTC
D441N-Rev	GAGATAGTACAAGTATTGGTTGATGAGTGGATTCATTAG
T491R-Fwd	CGACAACAACGTGTCTCAAGGACTGTGACTCAAAACAAC
T491R-Rev	GTTGTTTTGAGTCACAGTCCTTGAGACACGTTGTTGTCG
G530E-Fwd	CCAGCCACAAAGAAGAAGAGGACCGTTTC
G530E-Rev	GAAACGGTCCTCTTCTTCTTTGTGGCTGG
R533S-Fwd	CAAAGAAGGAGAGGACAGTTTCTTTCCTTTGTCTGG
R533S-Rev	CCAGACAAAGGAAAGAAACTGTCCTCTCCTTCTTTG
G549Q-Fwd	GGCAAACAAGGAACTCAAAGAGACAACGTGGATGC
G549Q-Rev	GCATCCACGTTGTCTCTTTGAGTTCCTTGTTTGCC
R550S-Fwd	CAAACAAGGAACTGGAAGCGACAACGTGGATG
R550S-Rev	CATCCACGTTGTCGCTTCCAGTTCCTTGTTTG
N552A-V553A-Fwd	GGAACTGGAAGAGACGCCGCGGATGCGGACAAAGTC
N552A-V553A-Rev	GACTTTGTCCGCATCCGCGGCGTCTCTTCCAGTTCC
D556P-Fwd	GAGACAACGTGGATGCGCCCAAAGTCATGATAACCAAC
D556P-Rev	GTTGGTTATCATGACTTTGGGCGCATCCACGTTGTCTC
I560F-Fwd	GATGCGGACAAAGTCATGTTCACCAACGAAGAAG
I560F-Rev	CTTCTTCGTTGGTGAACATGACTTTGTCCGCATC
N562Y-Fwd	CAAAGTCATGATAACCTACGAAGAAG
N562Y-Rev	CTTCTTCGTAGGTTATCATGACTTTG
T582Q-Fwd	CTATGGACAAGTGGCCCAAAACCACCAGAGTGCC

T582Q-Rev	GGCACTCTGGTGGTTTTGGGCCACTTGTCCATAG
Q588R-Fwd	CCACCAGAGTGCCAGGGCACAGGCGCAGACC
Q588R-REV	GGTCTGCGCCTGTGCCCTGGCACTCTGGTGG
Q588Y-Fwd	CCACCAGAGTGCCTACGCACAGGCGCAGACC
Q588Y-Rev	GGTCTGCGCCTGTGCGTAGGCACTCTGGTGG
A656Q-Fwd	CAAAAACACACCTGTACCTCAGGATCCTCCAACGGCCTTC
A656Q-Rev	GAAGGCCGTTGGAGGATCCTGAGGTACAGGTGTGTTTTTG
S669Q-Fwd	CAACAAGGACAAGCTGAACCAGTTCATCACCCAGTATTC
S669Q-Rev	GAATACTGGGTGATGAACTGGTTCAGCTTGTCCTTGTTG
N704Q-Fwd	GGAGATCCAGTACACTTCCCAGTATTACAAGTCTAATAATG
N704Q-Rev	CATTATTAGACTTGTAATACTGGGAAGTGTACTGGATCTCC
Y706D-Fwd	CAGTACACTTCCAACTATGACAAGTCTAATAATGTTG
Y706D-Rev	CAACATTATTAGACTTGTCATAGTTGGAAGTGTACTG
qLuc-Fwd	GCAAAACGCTTCCATCTTCC
qLuc-Rev	AGATCCACAACCTTCGCTTC
