# Supplementary material for: Structural characterization of antibody-responses following Zolgensma treatment for AAV capsid engineering to expand patient cohorts
Source: Nat Commun. 2025 Apr 19;16:3731. doi: 10.1038/s41467-025-59088-4 (PMC12009303; doi:10.1038/s41467-025-59088-4)
Supplement: Supplementary file 2 — Reporting Summary [file 41467_2025_59088_MOESM2_ESM.pdf]

nature portfolio

Corresponding author(s): Mario Mietzsch, Robert McKenna

Last updated by author(s): Apr 8, 2025

Reporting Summary

Nature Portfolio wishes to improve the reproducibility of the work that we publish. This form provides structure for consistency and transparency in reporting. For further information on Nature Portfolio policies, see our Editorial Policies and the Editorial Policy Checklist.

Statistics

For all statistical analyses, confirm that the following items are present in the figure legend, table legend, main text, or Methods section.

n/a	Confirmed	
☐	☒	The exact sample size (*n*) for each experimental group/condition, given as a discrete number and unit of measurement
☐	☒	A statement on whether measurements were taken from distinct samples or whether the same sample was measured repeatedly
☐	☒	The statistical test(s) used AND whether they are one- or two-sided<br>*Only common tests should be described solely by name; describe more complex techniques in the Methods section.*
☒	☐	A description of all covariates tested
☒	☐	A description of any assumptions or corrections, such as tests of normality and adjustment for multiple comparisons
☐	☒	A full description of the statistical parameters including central tendency (e.g. means) or other basic estimates (e.g. regression coefficient) AND variation (e.g. standard deviation) or associated estimates of uncertainty (e.g. confidence intervals)
☐	☒	For null hypothesis testing, the test statistic (e.g. *F*, *t*, *r*) with confidence intervals, effect sizes, degrees of freedom and *P* value noted<br>*Give P values as exact values whenever suitable.*
☒	☐	For Bayesian analysis, information on the choice of priors and Markov chain Monte Carlo settings
☒	☐	For hierarchical and complex designs, identification of the appropriate level for tests and full reporting of outcomes
☒	☐	Estimates of effect sizes (e.g. Cohen's *d*, Pearson's *r*), indicating how they were calculated

Our web collection on statistics for biologists contains articles on many of the points above.

Software and code

Policy information about availability of computer code

Data collection	The cryo-EM data was collected using the EPU software (Thermo Fisher).
Data analysis	The 3D image reconstructions were performed using cisTEM (https://cistem.org/) and Scipion (https://scipion.i2pc.es/). Atomic models were generated using Coot (https://www2.mrc-lmb.cam.ac.uk/personal/pemsley/coot/). The structures were visualized using Chimera (https://www.cgl.ucsf.edu/chimera/) and PyMol (https://pymol.org/).

For manuscripts utilizing custom algorithms or software that are central to the research but not yet described in published literature, software must be made available to editors and reviewers. We strongly encourage code deposition in a community repository (e.g. GitHub). See the Nature Portfolio guidelines for submitting code & software for further information.

Data

Policy information about availability of data

All manuscripts must include a data availability statement. This statement should provide the following information, where applicable:

- Accession codes, unique identifiers, or web links for publicly available datasets
- A description of any restrictions on data availability
- For clinical datasets or third party data, please ensure that the statement adheres to our policy

All atomic models and cryo-EM maps have been deposited to EMDB under the following accession numbers: 9B6N (Fab1-1), 9B6O (Fab1-2), 9B6P (Fab1-3), 9B6Q (Fab1-4), 9B6R (Fab1-5), 9B6S (Fab1-6), 9B6T (Fab1-7), 9B7K (Fab2-1), 9B7L (Fab2-2), 9B7M (Fab2-3), 9B7N (Fab2-4), 9B7O (Fab2-5), 9B7P (Fab2-6), 9B7Q (Fab2-7), 9B7R (Fab3-1), 9B7S (Fab3-2), 9B7T (Fab3-3), 9B7U (Fab3-4), 9B7V (Fab3-5), 9B7W (Fab3-6), 9B7X (Fab3-7) and EMD-44271 (Fab1-1), EMD-44272 (Fab1-2),

EMD-44273 (Fab1-3), EMD-44274 (Fab1-4), EMD-44275 (Fab1-5), EMD-44276 (Fab1-6), EMD-44277 (Fab1-7), EMD-44314 (Fab2-1), EMD-44315 (Fab2-2), EMD-44316 (Fab2-3), EMD-44317 (Fab2-4), EMD-44318 (Fab2-5), EMD-44319 (Fab2-6), EMD-44320 (Fab2-7), EMD-44321 (Fab3-1), EMD-44322 (Fab3-2), EMD-44323 (Fab3-3), EMD-44324 (Fab3-4), EMD-44325 (Fab3-5), EMD-44326 (Fab3-6), EMD-44327 (Fab3-7).

Research involving human participants, their data, or biological material

Policy information about studies with human participants or human data. See also policy information about sex, gender (identity/presentation), and sexual orientation and race, ethnicity and racism.

Reporting on sex and gender	Patient's data with respect to age and gender was de-identified.
Reporting on race, ethnicity, or other socially relevant groupings	n/a
Population characteristics	n/a
Recruitment	Sera samples and consent from healthy humans >18 years of age were obtained from the Australian Red Cross LifeBlood while patient samples were collected from SMA or MPSIII patients who had been genetically confirmed to have mutations in SMN1 or acetyl-CoA:alpha-glucosaminide N-acetyltransferas, respectively. Patients had either received or been precluded from AAV9 gene therapy trials as indicated in the text.
Ethics oversight	Human ethics approval was granted (LNR/18/SCHN/522) by the Ethics Committee of the Sydney Children's Hospital Network for the collection of analysis blood from patients and healthy controls.

Note that full information on the approval of the study protocol must also be provided in the manuscript.

Field-specific reporting

Please select the one below that is the best fit for your research. If you are not sure, read the appropriate sections before making your selection.

[x] Life sciences [ ] Behavioural & social sciences [ ] Ecological, evolutionary & environmental sciences

For a reference copy of the document with all sections, see nature.com/documents/nr-reporting-summary-flat.pdf

Life sciences study design

All studies must disclose on these points even when the disclosure is negative.

Sample size	Antibody sequences were derived from 3 patients, 7 antibodies per patient. This represents the largest set of human-derived monoclonal antibodies against the AAVs to date. The serology to determine endpoint titers were conducted twice. The animal study was conducted once. Transduction, neutralization, and dot-blot assays were performed in biological triplicate (n=3) to verify reproducibility. Three replicates in the standard procedure in our lab setting. Reproducibility of the experimental findings was confirmed.
Data exclusions	Cryo-EM micrographs of poor quality were excluded during 3D-image reconstruction. No other data was excluded.
Replication	The binding sites of the antibodies at high-resolution match the sites previously determined at low resolution (Logan et al. Structural and functional characterization of capsid binding by anti-AAV9 monoclonal antibodies from infants after SMA gene therapy. Mol Ther. 2023 Jul 5;31(7):1979-1993. doi: 10.1016/j.ymthe.2023.03.032). Each new capsid variant was initially tested as crude lysates and confirmed as purified AAV vectors.
Randomization	The 21 antibodies were derived from 3 patients but the reconstruction of each antibody-complex was analyzed independently.<br>Transduction, neutralization, and dot blot assay repeats were performed on different days.<br>Mice were randomly allocated across groups with even numbers of gender in each group.
Blinding	No patient information was provided for this study. Blinding was not necessary for serology as the relative differences for each sera was determined against two different AAV capsids and the null hypothesis was that there was no differences in seroreactivity to each AAV capsid. For the cryo-EM data collection blinding was not applicable. The structures were previously determined at low resolution.

Reporting for specific materials, systems and methods

We require information from authors about some types of materials, experimental systems and methods used in many studies. Here, indicate whether each material, system or method listed is relevant to your study. If you are not sure if a list item applies to your research, read the appropriate section before selecting a response.

Materials & experimental systems

n/a	Involved in the study	
	x	Antibodies
	x	Eukaryotic cell lines
x		Palaeontology and archaeology
	x	Animals and other organisms
x		Clinical data
x		Dual use research of concern
x		Plants

Methods

n/a	Involved in the study	
x		ChIP-seq
x		Flow cytometry
x		MRI-based neuroimaging

Antibodies

Antibodies used	21 recombinantly-expressed (in HEK293 cells) human antibodies. The specificity and generation of the antibodies were described in our previous study: Logan GJ et al. Structural and functional characterization of capsid binding by anti-AAV9 monoclonal antibodies from infants after SMA gene therapy. Mol Ther. 2023 Jul 5;31(7):1979-1993. doi: 10.1016/j.ymthe.2023.03.032. PMID: 37012705; PMCID: PMC10362397.<br>Goat Anti-Human-heavy IgG chain-HRP conjugated antibody (Sigma-Aldrich/Millipore AP309P) 1:10,000<br>anti-AAV VP1/VP2/VP3 mouse monoclonal, mAB-B1 (ARP 690058) 1:1000<br>Goat Anti-Human IgG H&L (HRP) (Abcam ab6858) 1:50000<br>anti-mouse-HRP (Cytiva NA931-1ML) 1:3000
Validation	The human antibodies are characterized in this study.

Eukaryotic cell lines

Policy information about cell lines and Sex and Gender in Research

Cell line source(s)	HEK293 cells were bought via ATCC.
Authentication	The ATCC bought cell lines were not authenicated.
Mycoplasma contamination	HEK293 cells were not tested for mycoplasma contamination.
Commonly misidentified lines (See ICLAC register)	n/a

Animals and other research organisms

Policy information about studies involving animals; ARRIVE guidelines recommended for reporting animal research, and Sex and Gender in Research

Laboratory animals	C57BL/6 mice, 8-weeks of age
Wild animals	n/a
Reporting on sex	Male and female mice were utilized
Field-collected samples	n/a
Ethics oversight	All animal care and experimental procedures were evaluated and approved by the Animal Care and Ethics Committee of the Children's Medical Research Institute and Children's Hospital at Westmead.

Note that full information on the approval of the study protocol must also be provided in the manuscript.

Plants

Seed stocks	n/a
Novel plant genotypes	n/a
Authentication	n/a
